# Balloon Mitral Valvotomy With the Accura Double-Lumen Balloon Catheter: Immediate and One-Year Clinical Outcomes

**DOI:** 10.7759/cureus.24610

**Published:** 2022-04-29

**Authors:** Suraj Khanal, Shrimanth Yamasandi Siddegowda, Basant Kumar

**Affiliations:** 1 Cardiology, Postgraduate Institute of Medical Education & Research, Chandigarh, IND

**Keywords:** rheumatic heart disease, mitral valve stenosis, balloon mitral valvotomy, complications, accura balloon

## Abstract

Objective

Despite the decline in the incidence of rheumatic heart disease in developed countries, the disease still remains endemic among individuals of low socioeconomic status. The aim of the study is to assess immediate and one-year outcomes of balloon mitral valvotomy using the double-lumen Accura balloon (Vascular Innovations Co., Nonthaburi, Thailand) in patients with mitral valve stenosis.

Methods

This was a single-centre, observational, investigator-initiated retrospective study. All consecutive patients undergoing balloon mitral valvotomy with the Accura balloon between January 2015 and June 2020 were included. The primary endpoint was procedural success defined as an increase in mitral valve area (MVA) ≥50% from basal valve area or final valve area of ≥1.5 cm^2^, in the absence of mitral regurgitation grade >2. Clinical, echocardiographic, and haemodynamic parameters were assessed at the one-year follow-up.

Results

A total of 62 patients underwent balloon mitral valvotomy. The mean age was 34.9 ± 8.0 years, and 54 (87.1%) patients were female. Mean Wilkins' echo score was 7.5 ± 0.5. Procedural success was achieved in 59 (96.7%) patients. Mean MVA increased from 0.75 ± 0.14 cm^2^ to 1.64 ± 0.21 cm^2^, and mean mitral valve gradient decreased from 24.9 ± 5.5 mmHg to 7.30 ± 1.40 mmHg. Atrial fibrillation, mitral valve replacement, and moderate to severe mitral regurgitation occurred in 36 (59.0%), two (3.3%), and two (3.3%) patients, respectively. No death, cerebrovascular accidents, restenosis, or redo procedures occurred.

Conclusion

Balloon mitral valvotomy using the double-lumen Accura balloon is safe in patients with mitral valve stenosis.

## Introduction

Rheumatic heart disease and antecedent rheumatic fever have almost been eliminated in affluent countries. However, in low- and middle-income countries, this disease remains endemic and mitral valve stenosis is one of the manifestations causing significant mortality and morbidity [1]. Historically, patients with symptomatic mitral valve stenosis were treated with closed surgical mitral commissurotomy. A few years later, with the advent of cardiopulmonary bypass, open commissurotomy replaced closed commissurotomy [2]. It was in 1984, when Kanji Inoue, a Japanese cardiac surgeon, inflated a diseased mitral valve using a balloon made of strong, yet pliant natural rubber, thus, the era of percutaneous balloon mitral valvotomy began [3]. Over the decades, percutaneous balloon mitral valvotomy has emerged as the preferred treatment for select patients with mitral valve stenosis with favourable anatomy. This is attributed to its ability to overcome complications inherent to surgical procedures whilst maintaining procedural efficacy [4].

Several earlier studies have been conducted to observe the outcomes of balloon mitral valvotomy. However, the follow-up has often been limited to weeks or months [5,6] or extended to 10 years [7,8] and beyond [9-13], not to mention the data that are somewhat redundant as these studies date back decades ago. Hence, there is a paucity of recent data documenting immediate and short-term outcomes of balloon mitral valvotomy. Thus, the present study attempted to fill this void in the literature and sought to determine immediate and short-term outcomes of balloon mitral valvotomy using the double-lumen Accura balloon (Vascular Innovations Co., Nonthaburi, Thailand) in patients with mitral valve stenosis in the current decade.

## Materials and methods

Study design and patient population

This was a single-centre, observational, investigator-initiated retrospective study. All consecutive patients undergoing balloon mitral valvotomy with the Accura balloon (Vascular Innovations Co., Nonthaburi, Thailand) between January 2015 and June 2020 were included. Patients with mitral regurgitation greater than moderate to severe, extensive mitral commissural calcification, left atrial (LA) thrombus on transesophageal echocardiography performed prior to the procedure, or severe aortic valve disease were excluded. This study adhered to the principles of the Declaration of Helsinki [14]. All patients provided informed consent for the procedure, and subsequent data collection and analysis for research purposes were conducted, which is the practice of the hospital, irrespective of any study to be conducted in future.

Accura balloon

**Figure 1:**
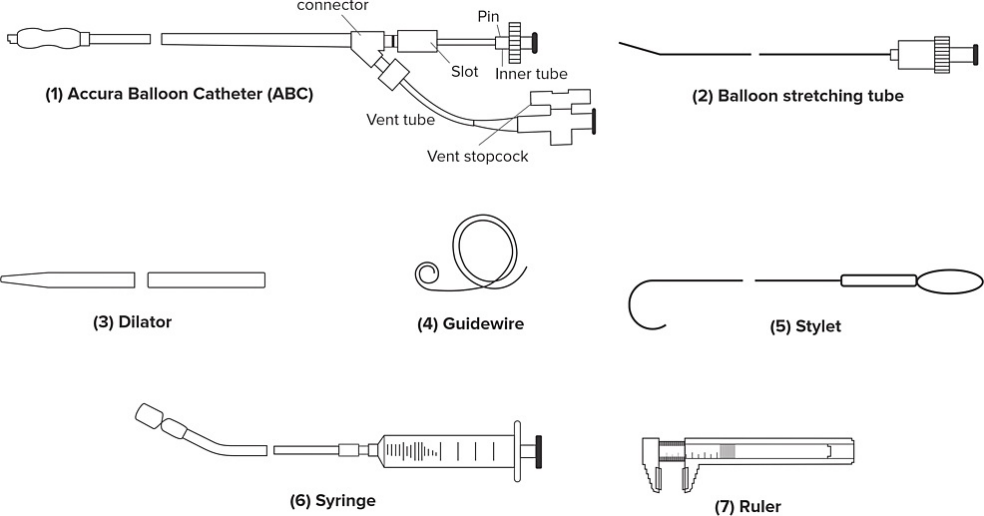
Figure showing Accura balloon and its components

**Figure 2:**
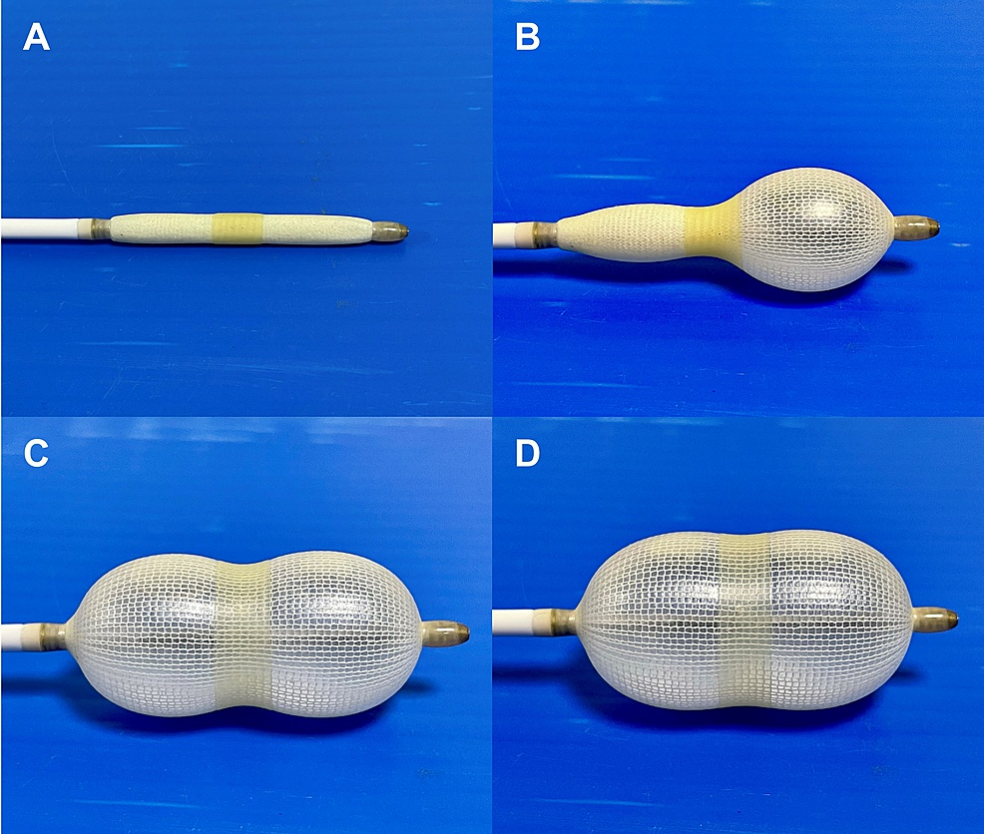
Accura balloon (A) Unexpanded balloon; (B) distal balloon inflation; (C) proximal balloon inflation; and (D) expansion to required size.

The Accura balloon is a double-lumen balloon catheter made of polyvinyl chloride with a balloon attached to the distal end. The balloon is formed of a double layer of latex with a third nylon mesh layer between the latex layers. The latex layer is extremely compliant, whereas the nylon mesh regulates the balloon diameter, shape, inner pressure, and resistance against balloon rupture. The Accura balloon has accessories that are supplied separately. These include a balloon stretching tube, dilator, LA guidewire, stylet, syringe, and ruler as illustrated in Figure 1. It is a three-stage expandable balloon as displayed in Figures 2A-2D. The balloon catheter shaft is 12 French (F) and 80 cm in length. A medical-grade stainless steel balloon stretching tube is used to stretch the balloon catheter prior to insertion, while the 14F 80 cm dilator dilates the septum (puncture site). The LA guidewire and stylet direct the balloon catheter to the mitral valve. The syringe is used to manually inflate the balloon, whilst the ruler measures the balloon diameter. There are two ports: the central lumen and the inflation port. Notably, there is no hole on the outside of the outer latex layer, thereby ruling out chances of seepage of blood when the balloon is fully expanded. The balloon is available in maximal sizes 22, 24, 26, 28, 29, and 30 mm. The size of the balloon represents the maximum diameter of the waist of the fully inflated balloon, and the average range of dilation is 3 mm.

Procedure

All patients underwent detailed clinical and echocardiographic evaluation using either two-dimensional (2D) Doppler or colour flow imaging to assess the severity of mitral valve stenosis, valve morphology, and mitral regurgitation. Mitral valve area (MVA) was determined by transthoracic echocardiography with planimetry in the parasternal short-axis view and by continuous-wave Doppler using the pressure half-time method. Mitral valve thickness, leaflet mobility, valvular calcification, and subvalvular disease were assessed using Wilkins' echo score [15]. Each parameter was graded 1-4 with a maximum score of 16. Echocardiography was also performed 24 hr after the procedure and at one-year follow-up. Right femoral access was attained in all patients. Cardiothoracic surgery backup was available for all cases. Balloon mitral valvotomy was performed with a double-lumen Accura balloon using a standard antegrade transseptal technique in all cases.

Primary endpoint and study definitions

Primary success was defined as an increase in MVA ≥50% from basal valve area or final valve area of ≥1.5 cm^2^, in the absence of mitral regurgitation grade >2. Restenosis was defined as a loss of 50% of the initial gain in MVA.

Data collection and follow-up

All data including demographic, clinical, hemodynamic, and echocardiographic data of patients were collected from patient medical records. Clinical and echocardiographic follow-ups were performed after one year.

Statistical analysis

All data were analyzed with the IBM Statistical Package for Social Sciences for Windows, version 20.0. (Armonk, NY: IBM Corp., USA). Data were presented using descriptive statistical methods. Continuous variables were presented as mean ± standard deviation, and categorical variables were expressed as frequency and percentages. Student t-test was used for comparison.

## Results

Baseline demographics

A total of 62 patients underwent balloon mitral valvotomy. The mean age of the study patients was 34.9 ± 8.0 years. There were 54 (87.1%) females. Thirty-one (50.0%) and two (3.2%) patients were classified as New York Heart Association (NYHA) classes III and IV, respectively. Atrial fibrillation was present in 34 (54.8%) patients. Mean MVA was 0.75 ± 0.1 cm^2^. Mean mitral valve gradient was 24.9 ± 5.5 mmHg. Wilkins' echo score ≥8 was observed in 28 (45.2%) patients. The baseline demographics are detailed in Table 1.

**Table 1:** Baseline demographics

Variable	Patients (n=62)
Age, years	34.9 ± 8.0
Female	54 (87.1%)
New York Heart Association class	
II	29 (46.8%)
III	31 (50.0%)
IV	2 (3.2%)
Rhythm	
Sinus	28 (45.2%)
Atrial fibrillation	34 (54.8%)
Mitral valve area, cm^2^	0.75 ± 0.14
≤0.5	5 (8.1%)
0.5–1.0	51 (82.3%)
≥1.0	6 (9.7%)
Mitral valve gradient, mmHg	24.9 ± 5.5
Wilkins' echo score	7.5 ± 0.5
≤7	34 (54.8%)
≥8	28 (45.2%)

Procedural outcomes

**Figure 3:**
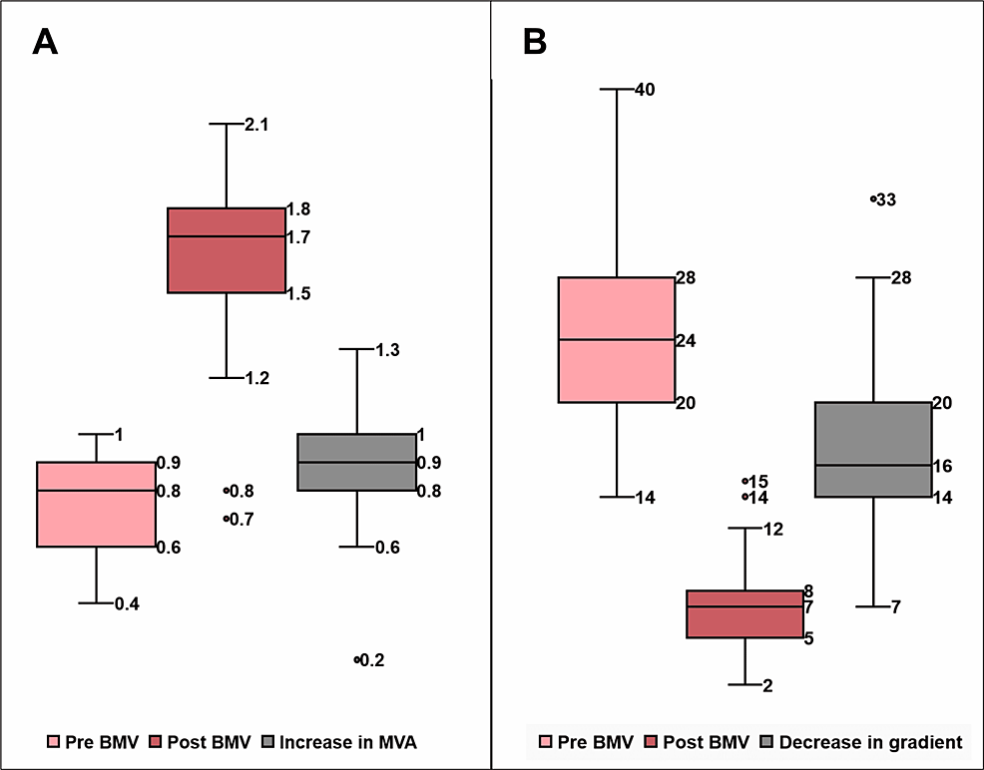
Box and whisker plot (A) Mean pre-balloon mitral valvuloplasty (BMV), post-BMV, and increase mitral valve area (MVA) and (B) mean pre-BMV, post-BMV, and decrease in mitral valve gradient following BMV

Balloon mitral valvotomy was performed in 61 (98.4%) patients. One patient suffered a cardiac tamponade due to a right atrium-inferior vena cava junction tear during the procedure and underwent successful emergency surgery for tear repair and mitral valve replacement. Procedural success was obtained in 59 (96.7%) patients. Two patients in whom balloon mitral valvotomy was unsuccessful had atrial fibrillation and greatly deformed mitral valves with areas of 0.6 cm^2^ and 0.5 cm^2^. Post-balloon mitral valvotomy mean MVA was 1.64 ± 0.21 cm^2^. Post-balloon mitral valvotomy mean mitral valve gradient was 7.30 ± 1.40 mmHg. Complications such as mitral regurgitation were observed in 28 (45.9%) patients. However, no other complications such as cerebrovascular accident, emergency mitral valve replacement, infective endocarditis, or death occurred immediately after the procedure. The procedural outcomes are demonstrated in Table 2, whilst the pre-balloon mitral valvuloplasty (BMV), post-BMV, and increase in mean MVA and pre-BMV, post-BMV, and decrease in mitral valve gradient are shown in Figures 3A, 3B, respectively.

**Table 2:** Procedural characteristics

Variable	Patients (n=61)
Balloon mitral valvotomy	61 (98.4%)
Procedural success	59 (96.7%)
Post-balloon mitral valvotomy mean mitral valve area, cm^2^	1.64 ± 0.21
Post-balloon mitral valvotomy mean mitral gradient, mmHg	7.30 ± 1.40
Final mitral valve area	
<1.5 cm^2^	11 (18.0%)
≥1.5 cm^2^	50 (82.0%)
Increase in mitral valve area, cm^2^	0.88 ± 0.15
Decrease in mitral valve gradient, mmHg	17.67 ± 4.78
Complications	
Mitral regurgitation	28 (45.9%)
Mild (grade 1)	15 (24.6%)
Moderate (grade 2)	11 (18.0%)
Moderate to severe (grade 3)	2 (3.3%)
Severe (grade 4)	0 (0%)
Cerebral vascular accident	0 (0%)
Emergency mitral valve repair	0 (0%)
Infective endocarditis	0 (0%)
Mortality	0 (0%)

One-year follow-up data

Follow-up data for clinical, echocardiographic, and hemodynamic variables were available for all 61 patients. At one-year follow-up, only one (1.6%) patient remained in NYHA class III/IV. Atrial fibrillation was observed in 36 (59.0%) patients. Two (3.3%) patients who had developed moderate to severe mitral regurgitation following the procedure showed no further worsening of mitral regurgitation. No patients required redo balloon mitral valvotomy or mitral valve replacement, suffered a cerebral vascular accident, or died during the follow-up period. One-year follow-up data are elaborated in Table 3. Furthermore, for the immediate and one-year results of the mean mitral valve area (1.64 ± 0.21 mm^2^ vs. 1.58 ± 0.31 mm^2^, p=0.292) and the mean transmitral gradient (7.30 ± 1.40 mmHg vs. 7.90 ± 3.50 mmHg, p=0.224), no statistically significant difference was observed.

**Table 3:** Follow-up at one year

Variable	Patients (n=61)
New York Heart Association class	
I	39 (63.9%)
II	21 (34.4%)
III/IV	1 (1.6%)
Atrial fibrillation	36 (59.0%)
Mitral gradient, mmHg	7.90 ± 3.50
Mitral valve area, cm^2^	1.58 ± 0.31
Redo balloon mitral valvotomy	0 (0%)
Mitral valve replacements	2 (3.3%)
Cerebral vascular accident	0 (0%)
Moderate to severe mitral regurgitation	2 (3.3%)
Mortality	0 (0%)

## Discussion

**Figure 4:**
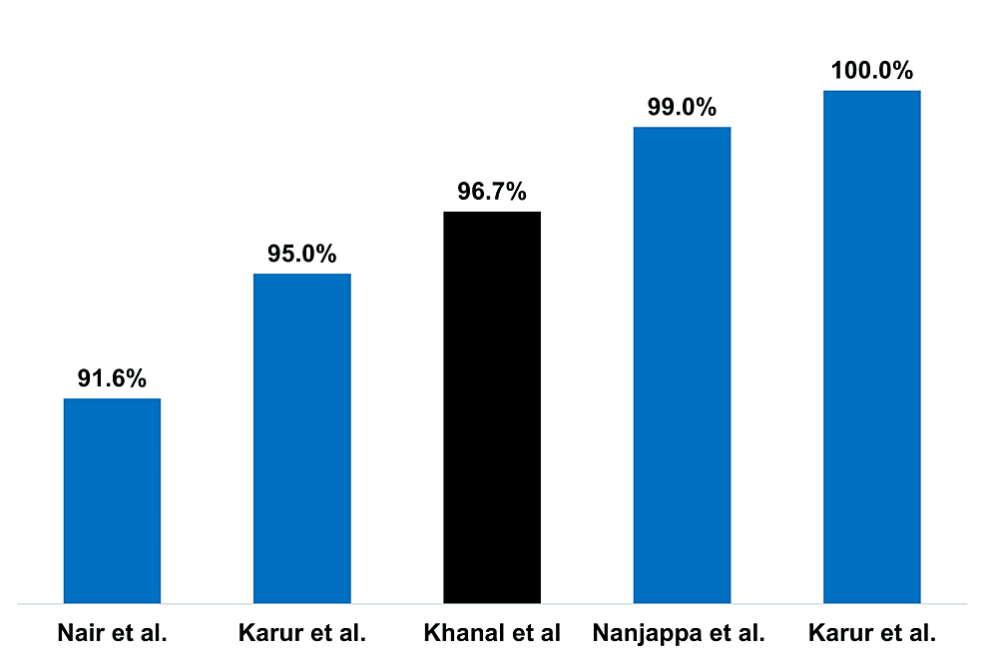
Procedural success rates of balloon mitral valvotomy using the Accura balloon Studies observed are as follows: Nair et al. [20], Karur et al. [22], and Nanjappa et al. [17].

The era of percutaneous balloon mitral valvotomy began in 1984. Throughout the decades, several operators have described their early experiences [16-19]. Thus, balloon mitral valvotomy has emerged as the standard of care for suitable patients with mitral stenosis. The present study sought to assess immediate and short-term outcomes of balloon mitral valvotomy using the double-lumen Accura balloon in patients with mitral valve stenosis in the current decade. Despite a modest sample size, study findings revealed a favourable procedural success rate of 96.7%. This finding is in line with procedural success rates observed over the preceding two decades. Success rates ranged from 79.5% to 100% [4,5,7-10,20-25]. Similarly, the success rate is in congruence with other studies [17,20,22] reporting outcomes with the Accura balloon as depicted in Figure 4.

Normal MVA values range from 4 to 6 cm^2^, but the majority of patients with MVA <1.5 cm^2^ are symptomatic. Hence, this cut-off value has been selected as the threshold for the definition of procedural success [11]. However, definitions vary marginally with respect to three specific criteria: mitral valve index or MVA as a cut-off value (mitral valve index ≥0.9 cm^2^/m^2^ or mitral valve area ≥1.5 cm^2^), mitral regurgitation (absent mitral regurgitation or severe mitral regurgitation grade <2), and in-hospital major adverse cardiac and cerebrovascular events (inclusion or exclusion as a criterion). These differing criteria may justify the vast range of procedural success rates observed in current literature. An earlier study [19] revealed that a post-procedural MVA of ≥1.5 cm^2^ predicts better long-term clinical outcomes after balloon mitral valvotomy, whilst another study [26] revealed a post-procedural balloon mitral valvotomy area of >1.75 cm^2^ yielded better intermediate outcomes. The present study defined procedural success as an increase in MVA ≥50% from basal valve area or final valve area of ≥1.5 cm^2^, in the absence of mitral regurgitation grade >2. However, of note, although the absence of adverse clinical events was excluded from the definition, adverse events such as cerebrovascular accidents, emergency mitral valve repair, or death did not occur. Mitral regurgitation was the only complication observed post-procedure, and this criterion has been included in the definition. This further reinforces the validity of the procedural success rate attained in this study. Moreover, two (3.3%) patients who had developed moderate to severe mitral regurgitation following the procedure showed no further worsening of mitral regurgitation at one-year follow-up.

The triple-lumen Inoue (Toray Industries, Tokyo, Japan) balloon and double-lumen Accura balloon are the currently available single balloon catheters for balloon mitral valvotomy. These balloons are formed of a double latex layer with a mesh layer between them. The balloon catheter design of Inoue and Accura differs to some extent [20]. The Inoue balloon has a vent in the balloon lumen and two small holes on the outer latex layer. The vent serves to remove air during preparation, whilst the holes are intended to avert deflation failure of the balloon. However, blood inevitably seeps in between the latex layers and embeds in the mesh layer. The vent and holes pose a great challenge when preparing, cleaning, and sterilizing the Inoue balloon. More critically, failure to clear all the blood from the vent or hole can lead to infection [5]. In contrast, the Accura balloon has no vent. However, the Inoue and Accura balloons have been shown to be similar in terms of safety, efficacy, and short-term outcomes: procedural success (Accura: 91.6% and Inoue: 93.6%), complications (Accura: 5.6% and Inoue: 6.6%), one-year restenosis (Accura: 1.6% and Inoue: 0.9%) patients, and one-year severe mitral regurgitation (Accura: 0.9% and Inoue: 1.6%) [20].

Over the three decades, since the inception of balloon mitral valvotomy, a few trends have been witnessed. The average cost per admission had increased significantly [27]. These costs are largely driven by the cost of the balloon [5]. The Inoue and Accura balloons vary vastly in terms of cost, a critical factor to be considered, especially as rheumatic mitral stenosis is endemic in low-income developing countries already under financial constraints. An earlier study [17] found that if all 912 cases were performed with new Inoue balloons, the cost would have been much less compared to if new Accura balloons had been used. Further reduction in costs stems from greater reusability of the Accura balloon (six times) than of the Inoue balloon (five times). A second study [20] conducted a few years ago reiterated the same findings. In developing countries, wherein the cost of the procedure is an imperative issue, the reuse of the balloon is almost necessary to reduce the cost of the procedure, again stressing the importance of ease of preparation, cleaning, and sterilization [20].

The second trend observed throughout the decades is the reduction in the volume of procedures performed in developing countries owing to the steady decline and almost eradication of rheumatic heart disease in affluent countries [10]. This aspect in combination with a simultaneous increase in age and comorbidities in patients has led to an increase in the complication rate [27]. In contrast, developing countries wherein rheumatic heart disease is endemic see a large number of cases being performed routinely. Such countries have a high operator volume and also a faster learning curve, resulting in fewer complications as reported in the present study. Increased operator has been associated with more favourable outcomes.

Study limitations

The major limitations were the modest sample size and the single-centre, retrospective design due to which the outcomes of the present study may not be generalized. The second limitation was the short follow-up duration. A longer follow-up duration is warranted to provide further insights into the outcomes of balloon mitral valvotomy. Future studies of larger sample sizes and longer follow-up duration in India to investigate this trend are warranted.

## Conclusions

Rheumatic heart disease still prevails in many developing countries. The present study explored the immediate and short outcomes of balloon mitral valvotomy using the double-lumen Accura balloon in patients with mitral valve stenosis. Study findings revealed a favourable procedural rate supporting the safety of the double-lumen Accura balloon. Moreover, these results yielded are at par with earlier studies. Nonetheless, further studies with a larger sample size and longer follow-up duration are warranted.
